# Cytoskeletal proteins in cortical development and disease: actin associated proteins in periventricular heterotopia

**DOI:** 10.3389/fncel.2015.00099

**Published:** 2015-04-01

**Authors:** Gewei Lian, Volney L. Sheen

**Affiliations:** Department of Neurology, Beth Israel Deaconess Medical Center and Harvard Medical SchoolBoston, MA, USA

**Keywords:** filamin, formin, RhoGTPases, actin cytoskeleton, proliferation, polarity, migration, periventricular heterotopia

## Abstract

The actin cytoskeleton regulates many important cellular processes in the brain, including cell division and proliferation, migration, and cytokinesis and differentiation. These developmental processes can be regulated through actin dependent vesicle and organelle movement, cell signaling, and the establishment and maintenance of cell junctions and cell shape. Many of these processes are mediated by extensive and intimate interactions of actin with cellular membranes and proteins. Disruption in the actin cytoskeleton in the brain gives rise to periventricular heterotopia (PH), a malformation of cortical development, characterized by abnormal neurons clustered deep in the brain along the lateral ventricles. This disorder can give rise to seizures, dyslexia and psychiatric disturbances. Anatomically, PH is characterized by a smaller brain (impaired proliferation), heterotopia (impaired initial migration) and disruption along the neuroependymal lining (impaired cell-cell adhesion). Genes causal for PH have also been implicated in actin-dependent processes. The current review provides mechanistic insight into actin cytoskeletal regulation of cortical development in the context of this malformation of cortical development.

## Introduction

The cerebral cortex originates from an expansion of neural tube, which consists of a single-cell-layered, pseudostratified neuroepithelial cells (neural stem cells; Götz and Huttner, 2005). Neuroepithelial cells extend their processes from the apical side (ventricular zone) to the basal lamina, and undergo interkinetic nuclear migration along the apical-basal axis throughout the cell cycle. The nuclei locate to the basal lamina during G1 phase, staying at the basal lamina during S phase, and transition back to the apical side during G2 phase. Mitosis of most neural stem cells occurs at the apical surface, where adhesion proteins like N-cadherin and cytoskeleton-associated proteins form dense and dynamic adhesion structures. As neuroepithelial cells divide, neural cells adopt different cell fate specification—one proliferating neural stem cells and another differentiating neuronal cells by symmetric and asymmetric divisions (Cremisi et al., 2003). At an early stage of mouse corticogenesis, the neuroepithelial cells mainly adopt symmetric cell divisions to expand the neuroepithelial plane, thereby promoting enlargement the ventricle surface. As development progresses, the neuroepithelial cells begin to be progressively biased toward asymmetric division to generate one self-renewed progenitor and another differentiating intermediate progenitors (basal progenitors) or post-mitotic neurons. The post-mitotic neurons attach and migrate along radial glial scaffolds out of the expanding ventricular zone (germinal zone) creating a new overlying layer of cells called the preplate. The laminar position of neurons is characteristic of their birthdate, such that younger neuroblasts migrate past their older counterparts to form the more superficial layers of the cortex. Finally, once neurons reach their cortical destinations, they differentiate and adopt the complex dendritic and axonal connections that are characteristic of fully mature cortical neurons (Ohnuma and Harris, 2003). At later stages, neurogenesis declines and astroglial differentiation begins.

While different actin cytoskeletal mechanisms are often independently implicated in regulation of neural proliferation, migration and differentiation (Duong et al., 1994; Schaar and McConnell, 2005; Munro, 2006; Witte and Bradke, 2008; Solecki et al., 2009; Moon and Wynshaw-Boris, 2013), a primary role for the actin cytoskeleton in facilitating crosstalk between various cell membrane and intracellular molecules would suggest that shared actin dependent pathways could mediate these developmental processes. For example, cell fate specification of neural stem cells along the neuroependymal lining depends upon the actin cytoskeleton to direct cytokinesis and localization of cell fate determining proteins. This actin dependent process dictates whether progenitors produce daughter progeny capable of self-renewal or postmitotic neurons (Knoblich, 2008). Under a constant rate of cell cycle, the greater the number of progenitors that undergo self-renewal, the larger the number of neural progenitors generated, giving rise to more post-mitotic neurons and leading to bigger brain size. Conversely, increased differentiation will lead to a fewer neural progenitors over time, and ultimately fewer neurons and smaller brain size. In this respect, a shared actin cytoskeletal dependent pathway regulates both neural proliferation and differentiation, thereby maintaining a delicate tune of self-renewal vs. differentiation to direct formation of the cerebral cortex. Within this same framework, the same actin cytoskeletal pathways that regulate cell fate specification will also mediate neuronal migration, as intermediate progenitors and post-mitotic neurons will migrate toward the intermediate zone or cortical plate respectively, whereas progenitors remain restricted to the ventricular zone. In this review, we focus on a fundamental actin cytoskeletal pathway that singularly directs each of these developmental stages during corticogenesis.

## Actin Cytoskeletal-Associated Proteins

The majority of actin cytoskeletal-associated proteins participate in the formation of actin filaments by regulating the dynamic processes of actin polymerization and de-polymerization. Actin polymerization occurs under the effect of actin-nucleating proteins, including Arp2/3 and formin proteins. The Arp2/3 complex nucleates G-actin in the lamellipodia to form short, branched actin filaments, whereas formins nucleate actin polymerization into long, unbranched filaments, which are important for both stress fiber formation and contractile ring assembly in mitosis (Bovellan et al., 2014; Fried et al., 2014; Khaitlina, 2014; Pan et al., 2014; Zhang et al., 2015). In addition, the actin polymerization state is also regulated by many small GTP-binding proteins like Rho GTPases, which cycle between active and inactive states through GTP to GDP exchange (Ciobanasu et al., 2013; Chen and Friml, 2014; Murali and Rajalingam, 2014). Actin polymerization coincides with a homeostatic de-polymerization of actin filaments through actin-severing molecules like cofilin and gelsolin, which bind and dissociate G-actin-GDP from actin filaments (Nag et al., 2013; Hild et al., 2014). Finally, the stability and contractile state of the actin cytoskeleton are regulated by many actin-crosslinking proteins, like filamins, actinin and myosin (Otey and Carpen, 2004; Ma and Adelstein, 2014; Modarres and Mofradt, 2014). Cooperative interaction between these various cytoskeletal-associated proteins can subserve multiple fundamental cell functions.

As F-actin-binding proteins, filamins are highly conserved and expressed in all eukaryotic cells. Filamins can crosslink actin filaments into orthogonal networks in the cellular cortical cytoplasm and participate in the anchoring of membrane proteins to the actin cytoskeleton (Gorlin et al., 1990). They also cross-link parallel stress fibers, thereby forming an aligned array in fibroblasts. Structurally, filamins are composed of a tandem calponin-homology (CH) actin-binding domain (ABD) at the N-terminus, 24 immunoglobulin-like repeat domains and a C-terminal dimerization domain. In vertebrates, filamins consist of three isoforms (FlnA, FlnB and FlnC) with molecular weights of ~280 kDa (Nakamura et al., 2011; Razinia et al., 2012). The *FLNA* gene is located on human chromosome X q28 and mouse chromosome X q37.89, encoding an approximate 2640-amino acid protein. FlnA is ubiquitously expressed in almost all the tissues, especially in developing brain. The *FLNB* gene resides on human chromosome 3 and mouse chromosome 14 and its encoded protein is predominantly expressed in bone, whereas FLNC is found in muscle tissue. Although FlnB and FlnC are expressed in the brain, they have not been clearly associated with neurological disorders (Sheen et al., 2002; Krakow et al., 2004; Okumura et al., 2013). FlnA, as a scaffolding protein, can interact with more than 45 proteins, including cell adhesion proteins (i.e., integrin) and cell cycle regulators. Recently, FlnA was found to be essential for formation of the E-cadherin-catenin adhesion complex (Feng et al., 2006; Ferland et al., 2009). FlnA also plays important roles in embryonic development by linking these important adhesion proteins and membrane receptors to the actin cytoskeleton. FlnA loss causes various tissue defects during embryonic development, including periventricular heterotopia (PH), skeletal malformation, disorders in vascular and cardiac development and intestinal defect (Fox et al., 1998; Sheen et al., 2001; Robertson et al., 2003). Missense mutations in *FLNA* gene, which are thought to lead to gain of function, are also associated with various human diseases, such as otopalatodigital syndrome, Melnick-Needles syndrome, thrombocytopenia, and intestinal pseudo-obstruction (Robertson et al., 2003). These roles for filamins in embryonic development are likely to be closely related to their functional mechanisms on diverse cellular processes in different types of cells, such as cell receptor signaling, endocytosis and exocytosis of membrane proteins through vesicle trafficking, and signaling transduction in the nucleus.

Formin(s) are members of a family of actin-nucleating, cytoskeletal-associated proteins, which stimulate actin nucleation at the barbed ends of actin to form linear filaments (Basu and Chang, 2007). The mammalian genome encodes more than 10 distinct formin proteins, such as mDia1-3, Daam1/2, and formin1/2. All the formin family of proteins includes two common domains, the formin homology 1 and 2 domains (FH1 and FH2). The FH1 domain contains a proline-rich sequence motif that can bind profilin and interact with certain proteins containing SRC Homology 3 (SH3) and WW domains. The WW domains are so named because of the presence of two conserved tryptophans (W) which are spaced 20–22 amino acids apart within the sequence (Bork and Sudol, 1994). Profilin-associated actin can be concentrated at the positive ends of actin filaments, and act as the major source of actin used for filament polymerization. The FH2 domain contains the actin-nucleating domain that can be associated with the fast-growing barbed end of actin filament and increase the actin polymerization rate by binding profilin/actin via its adjoining FH1 domains. The FH2 domain forms a ring-shaped dimer, which binds to the positive end of nascent actin filament and recruits two actin monomers for nucleation (Otomo et al., 2005). Flanking the FH1 and FH2 domains, the N-terminal GTPase-binding domain (GBD) and C-terminal autoregulatory domain (DAD) are also present in some subsets of formin proteins, and are important for formin activation (Gould et al., 2011). The binding of Rho GTPases to GBD is thought to open the DAD association with N-terminal autoinhibitory domain (DID), thereby changing the FH2 conformation and facilitating actin binding and polymerization. Except for the conserved FH2 domain, different subsets of formins display a distinct difference in other domain structures, which may endow formins with the capacity responding to varied sorts of cellular signals (Schönichen and Geyer, 2010). mDia 1-3 (Diaph1-3) genes are located in different genomic chromosomes, encode three 1100–1200 aa formin proteins carrying N-terminal GBD and DID domains, and C-terminal DAD domain. Loss of mDia proteins has been shown to disrupt apical adherens junctions, impact neuroepithelial polarity, and cause periventricular dysplasia in mouse and microcephaly in humans (Thumkeo et al., 2011). The* formin 1* (*Fmn1*) gene is located in mouse chromosome 2 and encodes a 1466-aa protein. It is predominantly expressed in the brain, kidney and developing limb buds, implying a potential role in development of various systems. The *formin 2* (*Fmn2*) gene is located on mouse chromosome 1 and encodes a 1578 aa protein. It is expressed in the central nervous system and developing mesenchyme, suggesting Fmn1 and Fmn2 may share some similar or common functions. Unlike the mDia proteins, it is not known whether Fmn1/2 contains GBD and DID domains. Collectively, expression of the formin genes in nervous system suggests that they may play diverse roles in neural cell adhesion, migration, proliferation and differentiation.

RhoGTPases are small GTP-binding proteins, the most commonly studied being RhoA, Rac and Cdc42 (Cook et al., 2014). They regulate many cell behaviors like polarity, adhesion, cell division and migration primarily by mediating actin cytoskeletal dynamics. Their regulation of actin polymerization is dependent on both Arp2/3- and formin1/2-nucleating processes. As master regulators of the cytoskeleton, RhoGTPases function between active and inactive states by exchanging GDP with GTP. The switch between activity states is modulated by three classes of regulatory proteins, referred to as guanine nucleotide exchange factors (RhoGEFs), GTPase activating proteins (RhoGAPs), and guanine nucleotide dissociation inhibitors (RhoGDIs). Although RhoA, Rac and Cdc42 all regulate actin remodeling and polymerization, they have distinctly different effects on actin reorganization through their respective downstream effectors. RhoA modulates actin polymerization, stress fiber assembly and focal adhesion formation through formins, Rock and myosin. Rac regulates actin filament polymerization through Arp2/3 and WAVE/WASP to produce lamellipodia and membrane ruffles at the leading edge. Cdc42 stimulates filament assembly and filopodia formation via WASP, Arp2/3 and formin. Collectively, RhoGTPases are expressed in neural cells during development of cerebral cortex, regulate downstream actin effectors (formins and Arps), and play pivotal roles in various neural cell developmental functions.

Periventricular Heterotopia is a malformation of cortical development, characterized by nodules of neurons ectopically located along the lateral ventricles. This disorder is thought to reflect impairments along several states of cortical development, including loss in neuroepithelial integrity, disrupted neural proliferation and cell fate specification, and impaired initial neural migration (Sheen, 2012). Mutations in the *FLNA* gene cause PH (Fox et al., 1998; Sheen et al., 2005). Filamins bind multiple cytoplasmic and cell surface receptors and molecules. Our work shows that filamins also bind formins and RhoGTPases, both of which have been implicated in some kinds of heterotopia formation (Thumkeo et al., 2011; Cappello, 2013). This trimeric complex provides a basic mechanism for modulation of broad actin cytoskeletal dependent processes, essential for cortical development and disease. Given the broad topic of cytoskeletal proteins in brain development, the current review focuses on filamin-associated proteins in corticogenesis, followed by their potential roles in causing PH.

## The Actin Cytoskeleton and Neuroepithelial Integrity

The neuroepithelium forms tight cell -cell or -matrix adhesion junctions at the apical surface of the cortical ventricle. The actin cytoskeleton is also assembled into dense actin cables along the apical surface and anchored onto these adhesion sites through cytoskeletal-associated proteins, such as filamin, formin and catenins. The apical lining is enriched for cytoskeletal proteins and actin filaments play determinant roles on the stability of adhesion junctions, as well as the polarity and integrity of the neuroepithelium. FlnA has been found to be essential for formation of the E-cadherin-catenin adhesion complex and its loss causes aberrant adherens junctions in multiple tissues (Feng et al., 2006; Ferland et al., 2009). Furthermore, FlnA-deficient neural progenitors exhibit poor adhesion to extracellular matrix proteins such as laminin. MDia1 and mDia3 formin proteins are also both expressed in the developing brain, and mDia3 is especially concentrated at the apical surface of the neuroepithelium. Loss of mDia1 and mDia3 impairs neuroepithelial cell polarity with attenuated apical actin belts and impaired apical adherens junctions (Thumkeo et al., 2011). Similar to the above findings, β-catenin is a cytoskeleton-associated protein, linking cadherin(s) to actin filament. Mice with conditional loss of β-catenin show several abnormalities in the neuroepithelium, including loss of adherens junctions, and impairment of radial migration of neurons toward the superficial layers (Machon et al., 2003). Finally, RhoGTPases such as RhoA and Cdc42 are highly expressed in neuroepithelium and essential for assembly and stability of apical actin cables. Conditional loss of RhoA and Cdc42 in central nervous system impairs apical localization of cadherin, apical accumulation of actin filament and cell-cell junctions. Collectively, these observations raise the possibility of a fundamental filamin-RhoGTPase-formin pathway in maintaining the location and function of adhesions molecules such as cadherins, which are required to ensure neuroependymal integrity.

## The Actin Cytoskeleton and Neural Progenitor Proliferation

Cytoskeletal-associated proteins may not regulate only the stability of cell-cell or cell-extracellular matrix adherens junctions, but also mediate cell proliferation by affecting cell cycle progression (Olson et al., 1995; Cappello et al., 2006; Woodhead et al., 2006; Katayama et al., 2011; Lian et al., 2012; Ercan-Sencicek et al., 2015). For example, actin regulates M (mitosis) phase progression, as the filaments are essential for cleavage furrow formation and completion of cytokinesis (Heng and Koh, 2010). Disruption of actin filaments by inhibitory agents such as latrunculin leads to cytokinesis failure due to a defect in the F-actin cable ring at the cleavage furrow (Lee and Song, 2007). FlnA shows strong expression in the cleavage furrow during mitosis. Additionally, functional loss of actin inhibits centrosome separation early in mitosis and leads to a delay in chromosome segregation late in mitosis (Rosenblatt et al., 2004; Cao et al., 2010). Finally, recent reports have suggested that the interaction of the cortical actin network with astral microtubules is crucial in establishing correct spindle orientation and in proper chromosome segregation in mammalian cells (Théry et al., 2005).

Prior to mitosis, G2-M phase entry requires remodeling of the actin cytoskeleton to change cell shape from an extended to a rounded morphology with cell retraction (Maddox and Burridge, 2003; Cao et al., 2010; Heng and Koh, 2010).This change in cell morphology initially requires activation of RhoA, which triggers a signaling cascade through formin and Rock to re-organize the actin cytoskeleton. Additionally, the cyclin dependent kinase 1 (Cdk1) promotes G2-M transition, and activated Cdk1 phosphorylates Rho GTPase activating protein (p190RhoGAP), down regulating p190RhoGAP hydrolysis of Rho-GTP and thereby promoting RhoA function (Maddox and Burridge, 2003). Our recent study shows that FlnA also regulates Cdk1 activity through Cdk1 phosphorylation and cyclin B degradation (Lian et al., 2012). Lastly, FlnA regulates RhoA activity through mediating p190RhoGAP accumulation in lipid rafts (Mammoto et al., 2007). Therefore, a potential signaling pathway underlying rearrangement of the actin filaments in G2-M phase involves a cascade beginning with FlnA, Cdk1, and p190RhoGAP, which then collectively mediate RhoA and formin/Rock function. This signaling pathway may also be crucial for cell cycle progression through M phase. Loss of FlnA impairs degradation of cyclin B1-related proteins, thereby delaying the onset and progression through mitosis (Lian et al., 2012). Furthermore, loss of FlnA increases the inhibitory phosphorylation of Cdk1 via its interaction with the kinase Wee1. In developing cerebral cortex, FlnA loss causes a decrease in proliferation rate of neural progenitors and a decline in neural progenitor pool size (Lian et al., 2012). This prolongation in cell cycle would relate to the impairment in actin filament rearrangement. Our understanding of the formin role in cell division is more limited, but insight can be gained from our understanding of formin function in other organ systems. Apart from a known interaction with filamins, Fmn1-deficient mice exhibit a reduction in digit number as well as the absence of a fibula due a defect in chondrocyte proliferation (Zhou et al., 2009). Fmn1 loss is linked to up-regulation of BMP and Msx1 but down-regulation of Fgf4 signals within the apical ectodermal ridge, which may influence mitosis. Finally, other RhoGTPase-related proteins also influence M phase progression. Both constitutively active and dominant negative Cdc42 inhibit cytokinesis (Drechsel et al., 1997), and loss of its downstream effector mDia3 causes chromosome misalignment during metaphase (Yasuda et al., 2004). Expression of dominant negative Rac1 retards adventricular nuclear migration, and promotes cytokinesis failures (Michaelson et al., 2008; Minobe et al., 2009).

The actin cytoskeleton mediates G1 phase progression after completion of mitosis. Disruption of actin polymerization by the cytochalasin D causes G1 phase arrest (Bohmer et al., 1996; Lian et al., 2012). The cytoskeletal-dependent effects on G1 progression is mediated through cyclin expression and cyclin-dependent kinase (Cdk) activation. More specifically, the actin cytoskeleton is required for anchorage-dependent expression of cyclin D1, activation of Cdk4/6, phosphorylation of the retinoblastoma protein and transition of G1 phase in non-transformed primary cells. In addition to this primary pathway, cytoskeletal-associated proteins regulate G1 phase progression through other secondary mechanisms. Inhibition of Cdc42, Rac1 and RhoA block G1 phase transition and serum-induced DNA synthesis (Olson et al., 1995; Leone et al., 2010). Active RhoA increases the expression of Skp2 protein, which promotes ubiquitinylation-dependent degradation of the Cdk inhibitor p27^kip1^ (Mammoto et al., 2004). Conversely, RhoA inactivation results in higher levels of p27^kip1^, thereby arresting cell cycle in G1 phase. RhoA inactivation or F-actin disruption are also shown to slow down the degradation of another Cdk inhibitor p21^Waf/Cip1^ (Coleman et al., 2006). With respect to filamins, our prior study suggests that FlnA also regulates neural progenitor proliferation in G1 phase (Lian et al., 2012) and directs cadherin-catenin complex formation. β-catenin is known to mediate G1 phase progression and neural proliferation, and tethers cadherin to the actin cytoskeleton (Woodhead et al., 2006). Conditional deletion of β-catenin in developing mouse brain results in a dramatic defect in neural progenitor proliferation and severe brain malformation. In contrast, overexpression of a stabilized β-catenin causes a significant increase in neural progenitor number and massive expansion of the cerebral cortex (Chenn and Walsh, 2002). Similar to filamins, formins may play coordinative effects on cell proliferation through G1 phase. Our ongoing studies suggest that loss of filamin and formin can affect β-catenin translocation and cyclin D expression. Compared to loss of FlnB or Fmn1 alone, loss of both Fmn1 and FlnB in mice leads to a more severe reduction in body size, weight and growth plate length (Hu et al., 2014). These findings would suggest that these actin associated proteins can mediate cell proliferation in multiple organ systems.

From the discussion above, the filamin-formin-RhoGTPase pathway can potentially be implicated in several phases of the cell cycle through interactions with various cell cycle associated proteins. A prevailing role for these proteins in regulation of actin dependent vesicle trafficking would provide a common mechanism for the transport and degradation cell cycle and cell fate proteins, which oversee neural proliferation.

## The Actin Cytoskeleton and Cell Polarity and Fate Specification

The neuroepithelium comprises a distinctive epithelial structure with apical-basal polarity. The actin cytoskeleton is selectively concentrated at the apical side along the ventricle of the developing cerebral cortex, forming a dense and dynamic filament belt to support tight adhesive junctions, cilium stability, and cell polarity, and to maintain a membrane barrier. Acting as regulators of cell shape and binding-partners for polarity proteins, the actin filament and its associated proteins are of key importance for regulating cell polarity (Ohno, 2001; Sawin, 2002; Etienne-Manneville, 2004; Witte and Bradke, 2008; Wang et al., 2009; Gonzalez-Billault et al., 2012). As an example, Rho-GTPases like RhoA, Cdc42 and Rac1 regulate actin cytoskeleton remodeling, focal adhesion formation and cell polarity (Etienne-Manneville, 2004; Iden and Collard, 2008; Gonzalez-Billault et al., 2012). Activated Cdc42 forms a stable hetero-tetrameric complex with polarizing proteins Par3, Par6, and atypical protein kinase C (PKCζ) and recruits these molecules to the leading edge to guide the reorientation of the microtubule and centrosome (Ohno, 2001; Etienne-Manneville, 2004). Cdc42-deficient neural progenitors exhibit multiple apical polarity-related defects including disorientation of cell division, aberrant location of the Par complex and adherens junctions, and severe impairments in the extension of nestin-positive radial fibers (Chen et al., 2006; Peng et al., 2013). Further, Cdc42 loss also causes PH and holoprosencephaly. As effectors of Rho-GTPases, formins and non-muscle myosin II have been shown to be indispensable for cell polarity (Habas et al., 2001; Ma et al., 2007). Depletion of the formin homologous protein Daam1 prevents Wnt/Fz activation of Rho and planar cell polarity during Xenopus gastrulation. Ablation of non-muscle myosin II-B in mice results in loss of neuroepithelial adhesion and severe hydrocephalus. Upstream of Rho GTPases, the association of FlnA with Wnt co-receptor Ror2 is required for Wnt5a-induced JNK activation, appropriate orientation of the microtubule organizing center and cell polarity (Nomachi et al., 2008). Further, loss of FlnA leads to a transition from bipolar neuron to multipolar neuron, suggesting a FlnA effect on neuronal polarity (Nagano et al., 2004). Given their physical interaction, filamins and RhoGTPases might regulate formin dependent polarized actin nucleation. Polarized actin provides a mechanism for establishment of neuroepithelial polarity.

Cytoskeletal proteins regulate cell proliferation not only by affecting cell cycle progression, but also through their control over cell fate specification (Chenn and Walsh, 2002; Taverna et al., 2014). During neuroepithelial cell fate specification, neural stem cells must undergo a decision process to undergo self-renewal or differentiate into intermediate progenitors or neurons. This process is dependent upon the asymmetric inheritance of cell fate determining proteins, which is regulated by polarized actin, actin dependent trafficking and degradation of cell fate proteins. In this respect cytoskeletal proteins could indirectly mediate cell fate. Cell fate determinants like Par3, aPKC, and numb as well as stem cell niche molecules like integrin and cadherin all directly or indirectly interact with the actin cytoskeleton, such that cell fate is influenced by cytoskeletal dynamics (Guo et al., 1996; Barros et al., 2003; Cappello et al., 2006; Woodhead et al., 2006). Furthermore, conditional deletion of Cdc42 at different stages of neurogenesis in mouse telencephalon results in an immediate increase in basal mitoses and altered differentiation of neural progenitors. In mesenchymal cells, loss of p190RhoGAP, which inactivates RhoA activity through GTP hydrolysis, causes a concomitant up-regulation of RhoA activity and increase in myogenic differentiation (but decrease in adipogenesis) (Sordella et al., 2003). In conditional RhoA-deleted embryos, RhoA-deficient neural progenitor cells exhibit accelerated proliferation and reduction in cell-cycle exit, indicating a change in cell fate specification (Katayama et al., 2011). Prior studies have shown that FlnA can bind to RhoA and integrin. Some of our initial observations suggest that loss of FlnA impairs cell cycle exit, in part through disruption of spindle orientation of neural progenitors during mitosis. Moreover, FlnA interactions with formins which nucleate actin in a polarized fashion would provide a basis for asymmetric delivery and localization of cell fate determining proteins.

## The Actin Cytoskeleton and Neural Migration

Cell migration is a highly dynamic process involving cell adhesion, extension, protrusion of filopodia and lamellipodia at leading edge and formation of contractile structure at rear edge. All the events require the dynamic remodeling of actin cytoskeleton (Rottner and Stradal, 2011). A variety of literatures have reported that actin cytoskeleton and its associated proteins are essential for cell migration (Fox and Walsh, 1999; Ridley et al., 2003; Raftopoulou and Hall, 2004; Govek et al., 2005; Broussard et al., 2008). For instance, disruption of actin filaments with drug cytochalasin C in migrating cerebellar granule cells can completely block cell migration (Rivas and Hatten, 1995). Further, melanocytes lacking FLNA show defects in filopodia formation and abnormal surface blebbing, implying a necessary role for FLNA in promoting the assembly of the cortical actin network. Several types of FLNA-deficient cells like macrophages, melanocytes and Dictyostelium amoebae cells display profound defects in motility and chemotaxis (Cunningham et al., 1992; Cox et al., 1996). In contrast, re-expression of FLNA in the cells can rescue each of these phenotypes, further establishing the essential effects of FLNA on migration. FLNA also is concentrated at rear edge of migrating leukocytes, implying FLNA may execute some important functions on rear edge retraction via FLNA-crosslink-driven force (Ohta et al., 2006). Notably, recent studies using transgenetic FlnA-deficient mouse model result in some contradictory conclusions to FLNA effects on neural cell migration (Fox et al., 1998; Feng et al., 2006; Hart et al., 2006). FlnA loss does not affect *in vivo* migration of neural crest cells into neural-crest-derived tissues like endocardial cushion, and the membrane ruffling, locomotion and migration of *in vitro* cultured fibroblast cells also appear normal. However, BrdU pulse labeling in embryonic day 15 null FlnA brains shows that the migration of BrdU^+^ cells into the cortical plate is slower than that of age matched wild type cells. Most BrdU^+^ cells from null FlnA cerebral cortices migrate into intermediate zone, but not into the cortical plate, as seen in their wild type littermates by 3 days post-labeling (Zhang et al., 2013). Further, cultured neural progenitor cells from E13 FlnA-null cerebral cortex also show poor spreading on laminin-coated coverslips, implying impairment in cell adhesion on extracellular matrix (Zhang et al., 2013). Finally, FlnA regulates the stability and turnover of adhesion and migratory associated proteins such as paxillin (Zhang et al., 2012, 2013). These migratory defects may in part be related to FlnA interactions with Filip, which regulates the degradation of the actin binding protein and thereby influences neural migration and cell specification (Nagano et al., 2002, 2004). These findings would suggest that filamins may regulate neural motility but do not completely abolish the capacity of progenitors to migrate to their intended site. Thus, these cytoskeletal associated proteins may not form the primary mechanism required to allow cells to migrate, but rather mediate processes (i.e., trafficking of particular receptors/molecules) that influence the rate at which neural progenitors move.

RhoGTPases such as RhoA, Rac1 and Cdc42 regulate remodeling of actin cytoskeleton, thereby serving as key regulators for cell morphology and migration (Raftopoulou and Hall, 2004). Their effects on neural migration have been extensively studied. Basically, Cdc42 and Rac1 stimulate formation of filopodia and lamellipodia (Yang et al., 2012), to direct neurite outgrowth and promote neural cell migration (Chen et al., 2007), whereas RhoA promotes retraction of rear edge and nuclear translocation of neural cell but impairs neurite extension. Other small GTP-binding proteins such as Rnd2 and Rnd3 have also been implicated in neural migration, although their relationship with filamins and formins is not known (Heng et al., 2008; Azzarelli et al., 2014). As downstream effectors of Rho GTPases, formins play potential important roles in polymerization and remodeling of actin filament. For instance, mDia deficiency impairs tangential migration of cortical and olfactory inhibitory interneurons (Shinohara et al., 2012). mDia-deficient neuroblasts exhibit reduced separation of the centrosome from the nucleus, retard nuclear translocation and concomitantly impair F-actin movement and condensation at the cellular rear. In non-neural cells, mDia1 has been shown to regulate formation of stable actin filaments and turnover of focal adhesions. mDia1 deletion impairs focal contacts, and decreases lamellipodial thickness in migrating cells (Yamana et al., 2006). Other formins like Fmn1 and FLR also are shown to function in cellular migration (Zhou et al., 2009; Hu et al., 2014). Fmn1 protein is more similar in sequence to dishevelled-associated activator of morphogenesis-1 (Daam1). Mutations in mouse Fmn1 gene result in limb deformities and incompletely penetrant renal aplasia, and display altered cell protrusion at the leading edge, defective cell spreading, and less focal adhesions. Our recent findings show that formin(s) can physically interact with filamin(s) and they co-express in developing bone and brain tissues (Hu et al., 2014). Loss of both formin and filamin results in serve defects in cell proliferation and migration in developing mouse thoracic wall and brain, suggesting that filamin and formin play cooperative roles in cell proliferation and migration.

Cell migration is a complex and cooperative process with protrusion at cell leading edge and with concomitant retraction at rear edge. The molecular mechanisms underlying neural migration may include some signaling pathways from G protein-coupled receptors, tyrosine-kinase receptors, PI3K, MAPK and Rho GTPases and cytoskeletal reorganization (Witte and Bradke, 2008; Jurberg et al., 2014). Here, it needs to be underscored that these processes of signal transductions require directional and robust vesicle trafficking that cytoskeletal proteins regulate. For instance, directional vesicle trafficking from cell rear edge to leading edge may play crucial roles on cell migration. Thus in a manner similar to establishment of cell polarity by directing the localization of fate determining proteins, filamins, formins and RhoGTPases can coordinate similar processes during neural migration.

The functional roles of actin cytoskeletal genes in adhesion junctions and cell migration are also mutually interdependent. In the developing cerebral cortex, radial glial cells extend their end feet onto the apical lining of ventricle, where they form dense cell to cell/matrix connections via cell adhesion proteins like cadherins and integrins at the tips of the end feet. Newborn neuron or pre-neuron migrate out of ventricle along the radial glial fibers. Thus, loss of cytoskeletal-associated proteins causes destabilization of the interdependent adhesion complex (adhesion proteins and actin filament) and disconnection of radial glial at the apical lining. Impairment in the connection of radial glial end feet would interrupt migration along the radial glial tracks, thereby contributing to the neuronal migration defect. In addition, actin cytoskeletal and adhesion proteins are also enriched within the growth cone of migrating neuron. Loss of these proteins and/or disruption of their trafficking likely influence neuronal migration itself.

Finally, neural migration is dependent on the differentiation state of the precursor. For example, Rac1 is necessary for neural progenitor transition from G1 to S phase, at least in part by regulating cyclin D levels and retinoblastoma protein phosphorylation. Loss of Rac1 in progenitors impairs the migration of ventral GABAergic neurons into the cortical plate. Ablation of Rac1 from postmitotic progenitors does not result in similar defects (Vidaki et al., 2012).

## Cytoskeletal-Associated Vesicle Trafficking: A Common Thread in Brain Development

Vesicle trafficking maintains the apical-basal polarity of neuroepithelium through directional vesicle transport and membrane protein sorting (transcytosis), thus underlying cell polarity, migration and asymmetric division (Rodriguez-Boulan et al., 2005). Vesicle trafficking includes endocytosis, exocytosis and endosomal recycling and sorting (Symons and Rusk, 2003). Endocytic trafficking is characterized by the internalization of plasma membrane and extracellular molecules via several distinct pathways: micropinocytosis, phagocytosis, and clathrin and caveolae-mediated endocytosis. This process is dependent on actin cytoskeletal-associated proteins like Rho GTPases (Ridley, 2006). For example, dominant-negative Cdc42 or Rac1 can block macropinocytosis, while constitutively active Cdc42 and Rac1 can restore the pinocytosis in immature dendritic cells (Garrett et al., 2000). Actin dynamics is also crucial for phagocytosis. Both Cdc42 and Rac participate in FcγR-mediated phagocytosis with Cdc42 directing pseudopod extension, and Rac functioning in pseudopod fusion and phagosome closure (Massol et al., 1998). During clathrin-mediated endocytosis, overexpression of constitutively active Rac1 and RhoA inhibits clathrin-mediated internalization of transferrin and EGF receptor in Hela cells (Lamaze et al., 1996), whereas RhoA can stimulate this process in polarized MDCK cells, suggesting that Rho GTPase function is dependent on cell polarity state (Leung et al., 1999). Caveolae are comprised of cholesterol-enriched internalized plasma membrane and are closely associated with actin filament. Integrins with caveolin have been shown to mediate RhoA activity in endothelial cells. Both RhoA and caveolin must co-localize and interact to mediate RhoA dependent actin remodeling (Nuno et al., 2009; Yang et al., 2011).

Formin and filamin proteins have been increasingly implicated in vesicle trafficking. MDia, together with myosin II, controls the initiation of E-cadherin endocytosis in the epithelium by regulating the lateral clustering of E-cadherin (Levayer et al., 2011). In oocytes, long-range transport of vesicles is regulated by Fmn2 through assembling an extensive actin network from the vesicles’ surfaces to plasma membrane. The vesicles move directionally along these actin cables to reach the cell surface (Schuh, 2011). Recent work also reveals that filamin binds to the caveolae marker, caveolin, and is required for endocytic trafficking of caveolin (Muriel et al., 2011). The endocytic trafficking of caveolae towards a recycling endosome is impaired in FLNA-deficient HeLa and M2-melanoma cells.

Besides the effect on endocytic trafficking, actin cytoskeletal-associated proteins may also be involved in exocytosis. Actin-associated proteins (filamin, myosins and Cdc42) are present in the Golgi complex, and actin filament may function as tracks for the myosin-driven movement of vesicles. Expression of Cdc42 mutants slows the exit of the basolateral marker N-cadherin from the trans-Golgi network while also stimulating the exit of the apical marker neurotrophin receptor p75 (Musch et al., 2001). RhoGTPases associate with the Golgi apparatus in an ARF-dependent manner. The *ADP ribosylation factor guanine exchange factor 2* (*ARFGEF2* encodes for BIG2) is a guanine nucleotide-exchange factor for ARF1/3, which play an important role in vesicular trafficking from Golgi complex to plasma membrane. Inhibition of BIG2 disrupts membranous localization of adherens junction protein E-cadherin and beta-catenin by preventing their transport from the Golgi apparatus to the cell surface, leading to PH formation (Sheen et al., 2004; Zhang et al., 2012, 2013). Given that filamin can physically bind and interact with ARFGEF2 (Zhang et al., 2012), filamin and its associated proteins might orchestrate the vesicle exocytosis from Golgi to plasma membrane.

## Genetics of Periventricular Heterotopia

Periventricular heterotopia is one of the most common malformation of cortical development and causes seizures, dyslexia, and psychiatric disturbances (Sheen, 2012). PH is characterized by bilateral ectopic neuronal nodules found along the lateral ventricles (Lu and Sheen, 2005). The nodules are caused by impaired migration from the ventricular zone and disruption in the integrity of the neuroependyma (Sheen, 2012). Mutations in the causative genes also cause microcephaly (meaning small brain) (Sheen et al., 2003, 2004).

The most common form of PH is inherited in an X-linked dominant fashion from mutations in the *FLNA* gene (Fox et al., 1998; Sheen et al., 2001, 2005). A second form of autosomal recessive PH with microcephaly (ARPHM) has been associated with mutations in the *ARFGEF2* gene. *ARFGEF2* encodes brefeldin-A inhibited guanine exchange factor-2 (BIG2) (Sheen et al., 2004). BIG2 is a protein kinase A anchoring protein (AKAP) which regulates Golgi-vesicle trafficking through its Sec7 domain. Recent work has identified cadherin receptor ligands in causing PH due to mutations in *FAT4* and *DCHS1* (Cappello et al., 2013).

Several mouse genes have been shown to cause PH formation and can be functionally linked. αSNAP is a SNARE-related protein, involved in vesicle fusion. Prior reports have shown that αSNAP mediates VE-cadherin localization through a β1-integrin-associated process (Andreeva et al., 2005). FlnA binds β1-integrin (Calderwood et al., 2001). Mekk4 binds and regulates FlnA (Chi et al., 2005), and therefore could indirectly regulate caveolin mediated endocytosis. The RhoGTPases bind FlnA and direct various aspects of intracellular actin dynamics, which are required for endosomal vesicle transport (Cappello et al., 2006; Katayama et al., 2011). Deficiency of the formin associated mDia disrupts integrity of neuroepithelium and causes periventricular dysplasia (Thumkeo et al., 2011). Similarly, Spred1 is a multidomain scaffolding protein that contains an ENA/VASP domain which modulates actin stress fiber remodeling, like filamins. Spred1 is also associated with specific endosomal vesicles (Phoenix and Temple, 2010). Finally, SCF-c-kit affects several downstream pathways including RAS/ERK and JAK/STAT pathways, both of which have been associated with Mekk4 and Spred1 activity (Rönnstrand, 2004; Soumiya et al., 2009).

Similar radiographic or anatomical findings of PH and potentially linked functions suggest that genes causal for PH might be involved in a common molecular pathway important in neural progenitor development. Prior studies have demonstrated a shared interaction between FlnA and Big2 in activating Arf to form vesicles at the cell membrane, and thereby regulate turnover/stability of cell adhesion molecules (Zhang et al., 2012, 2013).

## Anatomical Phenotypes Associated with PH

Periventricular heterotopia refers to heterotopic neurons along the lateral ventricles which are caused by impaired motility/migration and disruption of the neuroependymal lining. For example, loss of FlnA results in fewer post mitotic cells reaching the cortical plate compared to wild type (WT) following BrdU incorporation. Cells remain in the intermediate and ventricular zones, consistent with a cell autonomous migratory defect (Zhang et al., 2013). Disruption in the neuroependymal lining has also been reported with FlnA inhibition (Adams et al., 2012). Loss of neuroependymal integrity is the primary cause of heterotopia formation.

While human and mouse genes associated with PH show brain heterotopia, they all also regulate neural proliferation. Human *ARFGEF2* mutations cause microcephaly. While microcephaly is not seen in females with *FLNA* mutations (likely due to mosaicism), males die at birth and have been reported to have thinner cortices, and loss of cortical convolutions consistent with underlying microcephaly (Guerrini et al., 2004). Human mutations in cadherin-associated DCHS1 and FAT4 alter progenitor proliferation (Cappello et al., 2013). FlnA null mice also have microcephaly (Lian et al., 2012). Disruption of Cdc42 and RhoA cause PH in mice and lead to changes in brain size and/or progenitor proliferation (Cappello et al., 2006; Katayama et al., 2011). Dysregulation of PH associated *Napa*, *Stem Cell Factor 1* (*SCF*) and *Spred1* genes also alter progenitor proliferation in mice (Chae et al., 2004; Ferland et al., 2009; Phoenix and Temple, 2010). Mekk4 loss causes PH and a smaller brain with increased cell death (Chi et al., 2005). Formins have been implicated in proliferation of mouse neuroepithelial cells (Thumkeo et al., 2011). Lastly, a nonsense mutation of the formin related DIAPH1 (human mDia1) in humans has been found to cause microcephaly (Ercan-Sencicek et al., 2015).

There are several reasons to believe that the heterotopia formation, disruption in neuroependyma integrity, and impairments in neural proliferation are integrally linked. First, from an anatomical basis, FlnA loss leads to a smaller brain through prolongation in cell progression through mitosis (M) phase (Lian et al., 2012). In cortical development, M phase occurs at the neuroepithelial lining suggesting a shared mechanism with PH formation (defects in adhesion/migration also occur at the neurependyma). Second, from a molecular standpoint, we have shown that Big2/FlnA regulate turnover of adhesion molecules (catenins and cadherins) at the neuroependyma (Zhang et al., 2013). These molecules have been show to regulate brain size and their associated molecules cause PH (Chenn and Walsh, 2002; Cappello et al., 2013). Lastly, a delay in differentiation would also lead to a delay in neural migration into the cortical plate.

### Cellular and Molecular Mechanisms in PH

Periventricular heterotopia was initially thought to derive from a simple failure in neuronal migration given FLNA’s regulation of the actin cytoskeleton (Fox et al., 1998). However, several observations indicate that PH formation may be more complex, derived from diverse processes associated with cytoskeletal dynamics. First, BrdU labeling shows that loss of FlnA in developing mouse brain results in slower migration of neurons, but no PH formation, implying that the impairment in neuronal migration may not be the primary reason for PH formation (Zhang et al., 2013). Second, conditional deletion of RhoA in developing brain causes severe PH formation, but loss of RhoA does not impair neuronal migration (Katayama et al., 2011; Cappello, 2013). Third, as more genes causative for PH are identified, their shared function would argue that adherens junctions and actin cytoskeletal dynamics along the ventricular lining may be the primary pivotal factor for PH formation (Brault et al., 2001; Machon et al., 2003; Chae et al., 2004; Sheen et al., 2004; Kadowaki et al., 2007; Ma et al., 2007; Thumkeo et al., 2011; Peng et al., 2013). Consistent with this view are the findings that the actin filament network around the heterotopia in the PH brains is disrupted (Ferland et al., 2009). Concomitantly, the expression of neuroepithelial polarity and adherens junction proteins along the apical lining decreases or disappears.

Recent studies from this laboratory have begun to reconcile how two seemingly dissimilar proteins Big2 and FlnA can give rise to PH (Zhang et al., 2012, 2013). Either acute or chronic loss of either Big2 or FlnA leads to impairments in neural migration during development of the cortex. Migratory neural cells show defects in filopodia extension and attachment onto extracellular matrix coated surfaces. Both proteins physically bind and interact within neural progenitors, and loss of protein expression of either FlnA or Big2 leads to compensatory upregulation of the other. As with many proteins that FlnA binds, Big2 localization is dependent on phosphorylation of the actin binding protein which redirects Big2 from the Golgi to the cell membrane. Relocalization to the membrane allows Big2 to activate Arf1. Arfs have been shown to reside at the cell surface with ARF1 and ARF3 mediating endocytosis (Dong et al., 2010; Kondo et al., 2012). The reciprocal regulation likely reflects a negative feedback, where loss of Big2 promotes phospho-FlnA expression to enhance redistribution of Big2 to the membrane. Conversely, loss of FlnA enhances Big2 expression to allow for Big2 delivery to the membrane. These studies begin to suggest an integral relationship between FlnA dependent actin dynamics and Big2 dependent regulation of vesicle formation and trafficking.

### Periventricular Heterotopia as a Disorder of Vesicle Trafficking

**Figure 1:**
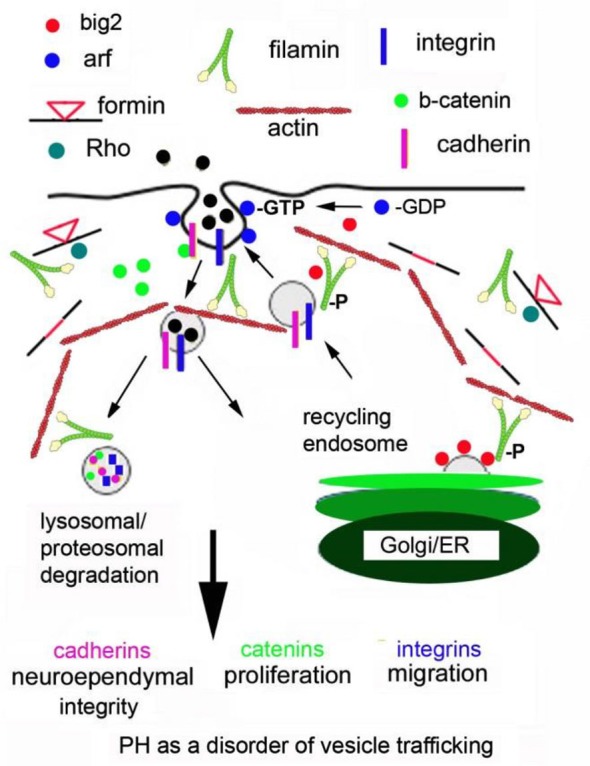
**Periventricular heterotopia as a disorder of vesicle trafficking.** FlnA phosphorylation localizes Big2 to the cell membrane, thereby activating the Arfs. Arfs are required for vesicle formation. Fmn interactions with FlnA are hypothesized to alter endocytosis and Fmn2 regulates vesicle trafficking and ultimately lysosomal degradation. Disruption of these processes leads to loss of cell-cell adhesion, impairing neuroepithelial integrity. Loss of focal adhesion sites causes defects in neural migration and impaired degradation of cell cycle associated proteins causes a reduction in neural proliferation.

Changes in Arf-dependent endocytosis have the potential to disrupt several cell developmental processes and provide a hypothetical model for PH formation (see the cartoon in Figure 1; Sheen, 2012). First, the primary anatomical defect leading to the PH phenotype (loss in neuroependymal integrity, impaired migration, and reduced proliferation) occurs within neural progenitors along the neuroepithelial lining. Genes implicated in PH (FlnA, Big2) regulate the stability, turnover and degradation of cell adhesion molecules (β-catenin, N-cadherin) and cell-ECM receptors (integrins, paxillin adaptor proteins). FlnA phosphorylation targets Big2 to the membrane, allowing for Arf-dependent activation and vesicle formation. Endocytosis occurs through a caveolin dependent mechanism leading to internalization of catenin/cadherin and integrins. Specificity for these molecules extends from their binding of filamins (Calderwood et al., 2001). Several associated PH genes (RhoA, αSNAP and Spred1) would be expected to participate in this process given their implied association with trafficking and/or endocytosis but their specific roles are not known. The downstream mechanisms that regulate endosomal and lysosomal/proteosomal processing in contributing to PH are also not known, although this review would point to a RhoA and Fmn dependent process.

In short, disruption of Big2 and FlnA (and presumably Fmn and RhoGTPases) would alter the stabilization, turnover and degradation of the cadherin, catenin and integrin proteins through impaired vesicle trafficking. Loss of cell adhesion (via altered cadherin) would disrupt the neuroependymal lining. Impaired cadherin stability would also alter catenin localization and function, thereby leading to impaired proliferation. Alteration of cell fate would be closely linked to proliferation and also effect neural migration. For example, slower progression through the cell cycle (as seen with loss of FlnA) causes delayed differentiation of neural progenitors at a given age (as seen with enhanced symmetric cell divisions from FlnA loss). This delayed differentiation would also lead to slower neural migration from the ventricular zone into the cortical plate. In this respect, many of the phenotypes seen with PH can be explained through changes in actin cytoskeletal regulation via FlnA-RhoA-Fmn2 (and Big2) within neural cells along the ventricular lining.

## Conclusion

Actin cytoskeletal-associated proteins play a variety of diverse and key roles in cerebral cortex development. A basic mechanism for filamins, formins and RhoGTPases may extend from their regulation of dynamic vesicle trafficking, in directed neural progenitor development. Directional transport of actin cytoskeletal-associated vesicles toward apicolateral or basolateral membranes along the neuroepithelium may be crucial for establishing polarity and maintaining adherens junctions. Loss of this function appears to be the primary pathology underlying PH formation. Cytoskeletal-regulated vesicle trafficking may transmit extracellular signals into cytoplasm and nucleus, as well as mediate degradation of cell cycle associated proteins, thereby regulating cell cycle progression and proliferation. PH has been associated with microcephaly. Finally, directional vesicle trafficking, as well as regulation of turnover of proteins toward the leading edge of migratory neural cells, would be required for proper neuronal migration, and account for the impaired migration seen in this disorder.

## Conflict of Interest Statement

The authors declare that the research was conducted in the absence of any commercial or financial relationships that could be construed as a potential conflict of interest.
